# Absence of a Role for Phosphorylation in the Tau Pathology of Alzheimer’s Disease

**DOI:** 10.3390/biom6020019

**Published:** 2016-04-08

**Authors:** Robert Y. K. Lai, Charles R. Harrington, Claude M. Wischik

**Affiliations:** 1 Medical Research Council Laboratory of Molecular Biology, Cambridge CB2 0QH, UK; robert.y.lai@btconnect.com; 2 TauRx Therapeutics Ltd. and School of Medicine, Medical Sciences and Nutrition, University of Aberdeen, Scotland AB25 2ZP, UK; c.harrington@abdn.ac.uk

**Keywords:** Alzheimer’s disease, tau protein, phosphorylation, protein aggregation

## Abstract

Alzheimer’s disease is characterized by redistribution of the tau protein pool from soluble to aggregated states. Aggregation forms proteolytically stable core polymers restricted to the repeat domain, and this binding interaction has prion-like properties. We have compared the binding properties of tau and tubulin *in vitro* using a system in which we can measure binding affinities for proteins alternated between solid and aqueous phases. The study reveals that a phase-shifted repeat domain fragment from the Paired Helical Filament core contains all that is required for high affinity tau-tau binding. Unlike tau-tubulin binding, tau-tau binding shows concentration-dependent enhancement in both phase directions due to an avidity effect which permits one molecule to bind to many as the concentration in the opposite phase increases. Phosphorylation of tau inhibits tau-tau binding and tau-tubulin binding to equivalent extents. Tau-tau binding is favoured over tau-tubulin binding by factors in the range 19–41-fold, irrespective of phosphorylation status. A critical requirement for tau to become aggregation-competent is prior binding to a solid-phase substrate, which induces a conformational change in the repeat domain permitting high-affinity binding to occur even if tau is phosphorylated. The endogenous species enabling this nucleation event to occur *in vivo* remains to be identified. The findings of the study suggest that development of disease-modifying drugs for tauopathies should not target phosphorylation, but rather should target inhibitors of tau-tau binding or inhibitors of the binding interaction with as yet unidentified endogenous polyanionic substrates required to nucleate tau assembly.

## 1. Introduction

Alzheimer’s disease (AD) is characterized by an almost complete redistribution of the tau protein pool from microtubule-bound to an aggregated state taking the form of pathological amorphous oligomers and paired helical filaments (PHFs) [1]. When the redistribution reaches a level sufficient to paralyse cytoskeletal transport through loss of microtubule function [2], cytoplasmic assemblies of PHFs become visible histologically as the neurofibrillary tangles originally discovered by Alzheimer, thereby giving the disease his name [3]. In clinico-pathological correlation studies, there is a close relationship between biochemical and histological measures of tau aggregation in post-mortem tissues and deterioration in mental functioning *ante mortem* [4], a correlation that holds from the earliest detectable stages of mental impairment [5]. More recently, the same correlation has been found to hold during life using positron emission tomography with ligands selective for aggregated tau [6,7]. It would appear plausible, therefore, that a treatment aiming to stop or slow the transfer of tau protein from the functional to the toxic aggregated state could be beneficial.

Defining an appropriate pharmaceutical target depends on having a better understanding of the molecular mechanism underlying this extensive redistribution of the tau protein pool. Many studies, as reviewed in [5], have shown that tau protein can form polymers *in vitro*, and it is assumed that any of these could be used to screen for tau aggregation inhibitors with potential therapeutic utility [8]. A common view holds that hyperphosphorylation of tau protein is a critical step in the aggregation cascade. This is based in part on a widely quoted misconception, which has entered the literature since the papers by Lee, Goedert and colleagues, that PHFs are composed “almost entirely of hyperphosphorylated tau protein” [9,10]. Phosphorylation of tau inhibits microtubule assembly [11] and phosphorylation at various sites of tau protein leads to small or major inhibitory effects on tubulin binding [12,13], that may be overcome by inclusion of appropriate osmolytes [14]. This has led to a hypothesis widely quoted in the literature that there is an imbalance between kinases and phosphatases which is disturbed in AD [15,16], leading tau protein to become detached from microtubules, and secondarily to aggregate. According to this scenario, a tau-based therapeutic approach would seek to target a kinase particularly responsible for a pattern of phosphorylation causing reduced microtubule stability. Two compounds, which were shown to reduce tau phosphorylation in cell-based and transgenic animal models, have been tested in phase 2 clinical trials. These trials were conducted in progressive supranuclear palsy, a rapidly progressive neurodegenerative disease characterized by pathological aggregation of tau protein, which is partly phosphorylated. Although the studies were of adequate size to demonstrate some degree of potential clinical benefit, none was shown (reviewed in [5]).

There are reasonably good theoretical reasons to have predicted these failures. We have argued elsewhere on structural and biochemical grounds that less than 5% of the tau molecules making up the PHF in AD is even N-terminally intact, let alone phosphorylated in domains often considered as defining the tau pathology of AD [17]. We have also shown previously that the build-up of soluble hyperphosphorylated tau that is supposed to be a precursor for PHF formation does not actually occur in the human brain until terminal stages of pathology. Whereas PHF formation can be detected as early as Braak stage 2 in the neocortex, the build-up of soluble hyperphosphorylated tau is not seen until Braak stage 6 [18], an interval of some 30 years. Thus, the tendency in the literature to conflate the concepts “phosphorylated tau” and “PHF tau” (e.g., [15,16]) is likely to be mistaken. Rather, it is more accurate to consider full-length phosphorylated tau as a “PHF-associated protein”.

It is important, therefore, to address the pathological relevance of the system used to identify potential therapeutic compounds. The assay we have chosen to develop appears useful in several respects. We have reported previously that a characteristic feature of the tau aggregation, which occurs in the AD brain is induction of a 15-residue conformational shift in the binding repeats. Thus, the N-termini of the tau fragments found in the proteolytically stable structural core of the PHF are located 15-residues C-terminal to the start of the repeats, and have a characteristic C-terminal truncation at position Glu-391, which is 15-residues C-terminal to the end of the repeats [19]. It is striking that this fragment and its homologues are nevertheless exactly 3 repeats in length, notwithstanding the phase shift. We have argued that this phase shift is the hallmark of an induced conformational transition in the repeat domain, which makes it possible to distinguish pharmaceutically between the normal tau-tubulin binding interaction, and the pathological tau-tau binding interaction, notwithstanding that both occur through the repeat domain [19]. The characteristic C-terminal truncation at Glu-391 creates a neo-epitope that can be detected with a monoclonal antibody (mAb) 423 raised against proteolytically stable PHF preparations [1,20]. In AD brain tissues, this neo-epitope is available without occlusion in early pathological tau oligomers, in tau oligomers accumulating in pathological lysosomes (so-called granulo-vacuolar degeneration), and in end-stage extracellular PHFs [21,22]. In intracellular PHFs, the epitope is occluded by a coating of full-length hyperphosphorylated PHF-associated tau which constitutes the proteolytically sensitive fuzzy coat of the PHF [23] and accounts for 5% of its mass [17]. The underlying neo-epitope can be exposed by treatment with formic acid [21,23,24,25].

The tau species making up the structural core of the PHF and its oligomeric precursors are derived from a mixture of the six isoforms of tau containing both 3 and 4 repeats (3R, 4R) derived from repeats 2/3/4 and repeats 1/3/4 of the 4R and 3R isoforms, respectively. The species derived from repeats 1/2/3 of the 4R isoform has an equivalent C-terminal truncation conferring on it an identical gel mobility when released from PHFs [26]. We have shown that these features represent the footprint of a pathological tau-tau binding interaction which locks the repeat domain into a characteristic proteolytically stable configuration, and that this binding interaction has prion-like properties *in vitro* [27]. This stabilised configuration propagates and amplifies the Glu-391 truncation through repeated cycles of binding of additional full-length tau and proteolytic digestion in a templated prion-like manner.

We have used the same experimental system to understand better the determinants of the tau-tau binding interaction of the structural core of the PHF. One particularly useful feature of this system is the ability to determine binding affinities separately in aqueous and solid phases. This is important, as it has been suggested that the kinetic barrier to tau aggregation can be overcome by a nucleation event of some kind [28,29,30,31,32]. We have sought to determine whether tau-tau binding is enhanced by providing full-length tau as a binding partner *versus* binding only via the core tau fragment of the PHF. Furthermore, we have been able to examine in detail the potential role of (hyper) phosphorylation of tau protein in driving pathological aggregation of tau protein in both aqueous and solid phases and to compare this with its effect on the tau-tubulin binding interaction using the same detection system. By these means, we have aimed to get a better understanding of the critical factors responsible for pathological aggregation of tau protein with a view to development of pathologically relevant drug screening assays for optimization of tau aggregation inhibitors.

## 2. Materials and Methods

*Preparation of Tau Protein.* Recombinant tau (htau40 (T40), the isoform with 2 N-terminal domains and 4 repeats in the microtubule-binding domain (2N4R)) and truncated dGA tau (amino acids 297–390 of T40) were prepared as described previously. Tau from rat brain or adult human brain was extracted with perchloric acid [33,34]. In brief, brain tissue was homogenized in 2.5% perchloric acid (4 mL/g brain tissue) in the presence of protease inhibitors and phosphatase inhibitors and left on ice for 20 min. It was then centrifuged at 13,000 × *g* for 10 min. The supernatant fraction was dialyzed against Tris-HCl (100 mM, pH 7.4) for 3 h at 4 °C, then against Tris-HCl (5 mM, pH 7.4) overnight at 4 °C. The dialysate was centrifuged at 13,000 × *g*. Soluble tau was collected in the supernatant fraction. Tau protein was also repurified, following hyperphosphorylation *in vitro*, prior to use in the binding assays. The nomenclature used for the various tau protein preparations is summarized in Table 1.

*Hyperphosphorylation of Tau by Rat Brain Extract.* Hyperphosphorylation of tau was carried out according to Biernet *et al.* [36] using a rat brain extract containing kinases. About 20 to 50 mg of tau protein (recombinant or extracted from brain tissue) was used in each phosphorylation reaction. Kinase buffer (A) contained 20 mM Tris-HCl, 10 mM EGTA, 4 mM DTT, 4 mm MgCl_2_, 4 mM ATP, 4 mM PMSF, 40 mg/mL pepstatin, 40 mg/mL leupeptin, 40 mg/mL aprotinin and 20 mM okadaic acid (adjusted to pH 7.4 with 3 M NaOH and 2 M HCl). The final [Na^+^] and [Cl^−^] was approximately 30 mM and 10 mM, respectively; excessive salt was found to inhibit kinase activity. Tau protein was phosphorylated by incubation of a reaction mixture containing 15 mL of tau protein (1–3 mg/mL), 16 mL of buffer A, and 1 mL of rat brain extract.

*SDS Gel Electrophoresis Immunoblotting and Immunoelectronmicroscopy.* Standard electrophoresis and immunoblotting procedures were used as described [23,33,34]. Immunoblots were developed either with horseradish peroxidase or the ABC kit (Vector Laboratories). The mAbs 7/51, 21/D10, 27/499 and 27/342 were used in the form of undiluted hybridoma culture supernatant fluids. The mAb AT8 (Innogenetics, Belgium) was used at 1/1000 dilution. The mAb 7/51 recognizes an epitope in the last tau repeat [20,27]; 27/499 recognizes a human-specific tau segment between residues Gly-14 and Gln-26 [27]; 27/342 recognizes a segment between residues Ser-208 and Pro-251 [27], and have been described previously. The mAb 21/D10 is an IgM antibody raised against the A68-tau brain extract [9] by us. Non-Pronase-treated PHFs were incubated with mAbs and immunolabeled with gold conjugated anti-mouse IgG (whole molecule) as described [23]. Samples were pretreated with alkaline phosphatase according to [35].

*Tau Binding Assay.* This was carried out, as described previously [27]. Solid-phase tau was coated on 96-well poly-(vinyl chloride) microtitre plates in 50 mM carbonate buffer at 37 °C for 1 h. The coating concentration was 0–20 mg/mL (0–1.7 μM). The plate was washed twice with 0.05% Tween 20 at room temperature, followed by blocking with 2% dried milk (Marvel) in PBST for 1 h at 37 °C. After washing twice with 0.05% Tween 20 at room temperature, aqueous-phase tau was added. This was diluted in 1% gelatin in PBST, at a concentration of 0–300 mg/mL (0–26 μM). Incubation lasted 1 h at 37 °C. The plate was washed twice with 0.05% Tween 20 at room temperature. mAbs 27/499 or 27/342, diluted with an equal volume of 2% Marvel in PBST, were incubated for 1 h at 37 °C. After washing twice with 0.05% Tween 20 at room temperature, horseradish peroxidase-conjugated goat-anti-mouse antibody (1/1000 in PBST) was incubated for 1 h at 37 °C. The plate was washed and incubated with substrate solution containing tetramethylbenzidine and H_2_O_2_ and the rate of change of absorbance measured using a V_max_ plate reader (Molecular Diagnostics, California) as described previously [37]. Each experiment was performed in triplicate. The experimental design included controls in which both solid-phase and aqueous-phase tau were omitted, and also with either one of the two omitted.

*Data Analysis.* This was performed as described previously [27]. The average readings for the rate of change of absorbance were plotted against the plating- or aqueous-phase concentrations of tau, and curves were fitted according to the Langmuir equation (formally equivalent to the Michaelis-Menten equation) with the Kaleidagraph (Synergy, Philadelphia) or SysStat (SPSS Inc., Chicago, IL, USA) programs using quasi-Newton approximation. All curve-fitting correlation coefficients were greater than 0.95.

## 3. Results

### 3.1. Properties of Antibodies Used to Measure Tau-Tau Binding Interactions

**Figure 1:**
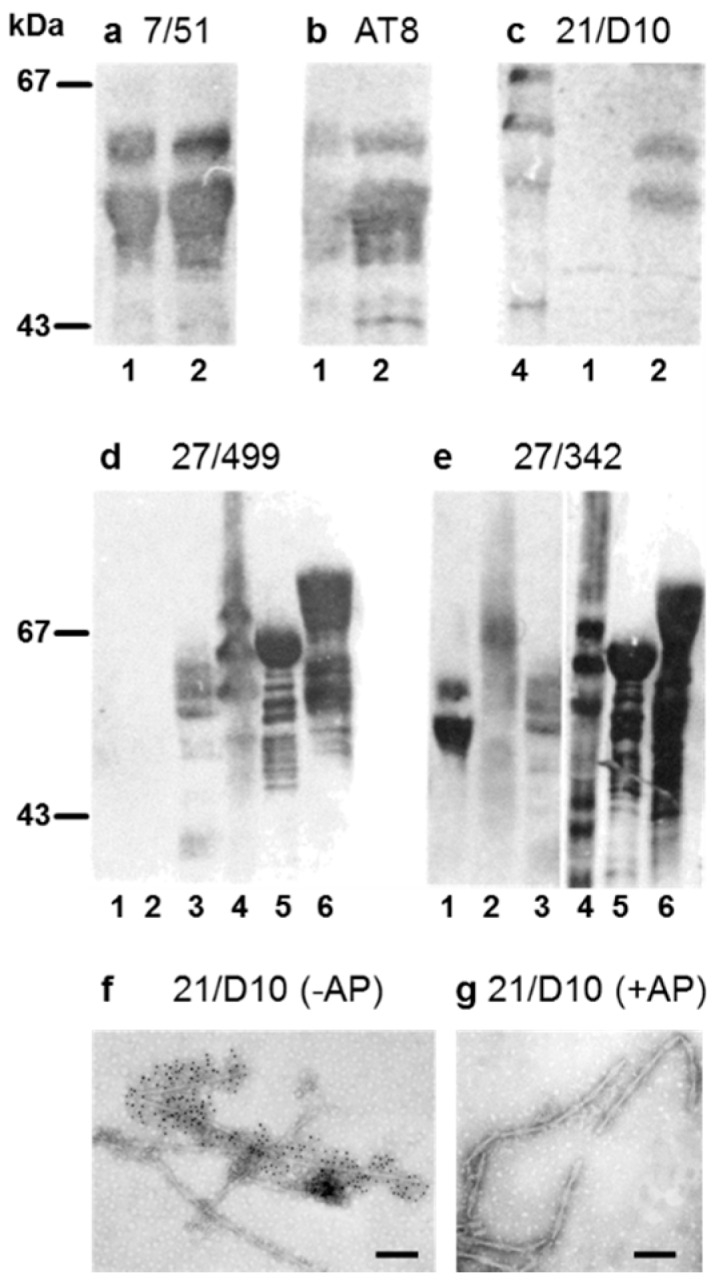
Immunoreactivity profile for tau antibodies. (**a**) The mAb 7/51 recognised tau proteins isolated from the brain of neonatal rat both before (lane 1; NT) and after (lane 2; NTP) hyperphosphorylation *in vitro*; (**b**) The mAb AT8 recognised endogenously phosphorylated tau (lane 1), the extent of which is increased following further *in vitro* phosphorylation (lane 2); (**c**) The mAb 21/D10 detected a phosphorylation-dependent epitope which is absent (lane 1) in endogenously phosphorylated neonatal tau (lane 1), but present following *in vitro* hyperphosphorylation (lane 2), and is also present in sarkosyl-insoluble tau protein isolated from AD brain tissue (lane 4); (**d**) The human tau-specific mAb 27/499 did not recognise neonatal tau isolated from rat before (lane 1) or after phosphorylation *in vitro* (lane 2), but recognised tau protein isolated from the brain of normal adult human (lane 3) and in the sarkosyl-insoluble tau extract from AD brain (lane 4), and the recombinant human tau both before (lane 5; T40) and after (lane 6; T40P) hyperphosphorylation *in vitro*; (**e**) In contrast, mAb 27/342 recognised neonatal rat tau both before (lane 1) and after (lane 2) hyperphosphorylation *in vitro*, but otherwise had a similar immunoreactive profile to mAb 27/499; (**f**,**g**) Non-Pronase treated PHFs from AD brain tissue were immunodecorated by mAb 21/10, as visualized by gold particles. The latter immunoreactivity was abolished by prior treatment of PHFs with alkaline phosphatase (AP).

We have used a combination of antibodies to different epitopes spanning the tau protein in order to measure different combinations of proteins in binding assays. The characterization of these antibodies on immunoblots is shown in Figure 1. The mAb 7/51 reacts with the central core domain of tau protein and the epitope is present in all isoforms of tau whether phosphorylated or not. The mAb AT8 was used as a marker for phosphorylated tau with its epitope dependent on phosphorylation of Ser-202 and Thr-205 [38]. We have generated a new antibody, mAb 21/D10 raised against hyperphosphorylated A68 tau proteins [31], which reacts with sarkosyl-insoluble tau protein isolated from AD brain tissue protein. The mAb 21/D10 detected a phosphorylation-dependent epitope which is absent in endogenously phosphorylated neonatal tau, but is present when neonatal tau has been further phosphorylated *in vitro* (Figure 1). Non-Pronase treated PHFs were decorated by mAb 21/D10 but immunoreactivity was lost following pretreatment with alkaline phosphatase (Figure 1f,g) or after proteolysis with Pronase (not shown), both conditions in which the structure of the filaments is retained. In addition to the altered mobility observed on SDS-PAGE and the increased amount, these phosphorylation-dependent antibodies confirm that the tau had been hyperphosphorylated *in vitro*, although the complete extent of phosphorylation of the many potential sites in tau was not characterized further. The kinase extract used is known to phosphorylate Ser-262 [12], a site that is effective in detaching tau from microtubules, but also one that is close to the repeat domain of the protein, and one that has been shown to prevent the aggregation of tau *in vitro* [39]. The mAb 27/499 recognises a human-specific epitope in the N-terminal fragment of human tau that differs notably from tau of other species such as mouse and rat. The mAb 27/499 did not recognise neonatal tau isolated from rat before or after phosphorylation *in vitro*, but recognised tau protein isolated from the brain of normal adult human, tau protein in the sarkosyl-insoluble extract from AD brain, and recombinant human tau before and after hyperphosphorylation *in vitro*. In contrast, mAb 27/342 recognised neonatal rat tau regardless of whether it had been hyperphosphorylated *in vitro*; it recognises an epitope that is N-terminal to the central core domain of tau (residues 208–251, [27]).

### 3.2. Solid-Phase Concentration-Dependent Enhancement of Tau Binding Affinity

A truncated repeat domain tau fragment corresponding to phase-shifted repeats 2/3/4 of the human 4R tau isoform (one of the 3-repeat species found in the core of the PHF, referred to as “dGA”, residues 297–390 and terminating at Ala-390 [34]) was first plated in the solid-phase (“adsorbed” or “ads” species). Full-length, non-phosphorylated recombinant human 2N4R tau (“T40”) was incubated in the aqueous-phase of the assay (“aqueous” or “aq” species). The nomenclature used is described in Table 1 and the data obtained for all binding constants defined below are summarised in Table 2.

**Figure 2:**
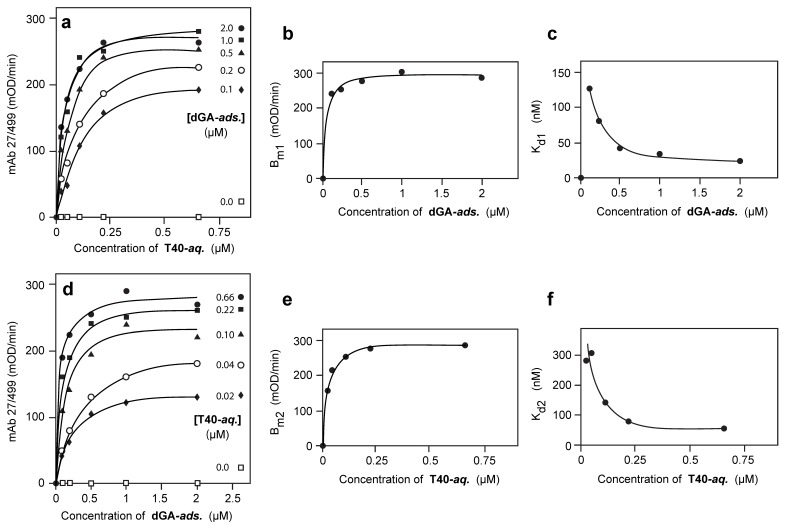
Tau-tau binding through the repeat domain. The truncated repeat domain dGA tau fragment was adsorbed (“ads.”) tothe solid phase at the concentrations indicated and binding of full-length T40 in the aqueous-phase (“aq.”) measured immunochemically using mAb 27/499. (**a**) For any fixed concentration of dGA-ads., binding of T40-aq. could be approximated by a family of Langmuir curves (Equation (1) in text). Systematic variation in the apparent B_max_ (B_m1_, (**b**)) and K_d_ (K_d1_, (**c**)) values depended on the concentration of dGA-ads. and could be approximated by the empirical relationships described by Equations (2) and (3) in the text. Conversely, for any fixed concentration of T40 in the aqueous-phase, binding could be approximated by a corresponding set of Langmuir curves (**d**) (Equation (4) in text). In this case, systematic variation in the apparent B_max_ (B_m2_, (**e**)) and K_d_ (K_d2_, (**f**)) values depended on the concentration of T40 in the aqueous-phase and could be approximated by the empirical relationships described by Equations (5) and (6) in the text. Correlation coefficients for all approximations exceeded 0.94.

As seen in Figure 2a, for any fixed plating concentration of adsorbed dGA, the binding data for aqueous T40 detected by N-terminal tau immunoreactivity (mAb 27/499) can be approximated by the standard relationship:

*B = B_m1_·[T40]/(K_d1_ + [T40])* (1)

The values of the constants *B_m1_* and *K_d1_* were found to vary systematically with the concentration of dGA in the solid phase, according to the following empirically derived relationships:

*B_m1_ = P_1_·[dGA]/(Q_1_ + [dGA])* (2)

*K_d1_ = (M_1_/[dGA]) + N_1_* (3)

**Figure 3:**
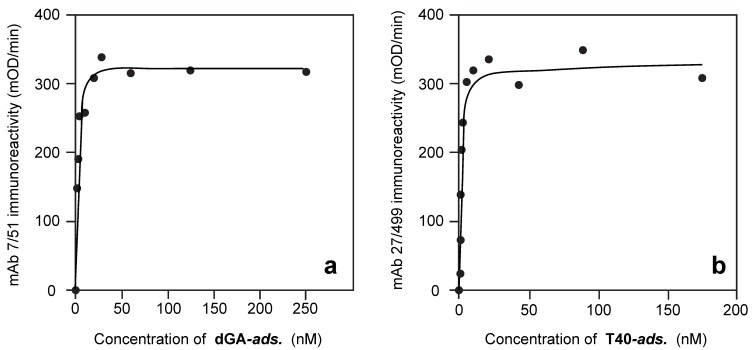
Non-specific binding of dGA (**a**) or full-length tau (**b**) to the solid-phase PVC matrix. Binding was detected using mAbs 7/51 or 499, respectively. In both instances, saturation of non-specific binding sites occurred at plating concentrations greater than 20 nM.

These relationships, depicted in Figure 2b,c, respectively, demonstrate that the apparent B_max_ values increase, and the apparent K_d1_ values decrease, with increasing plating concentrations of adsorbed dGA, approaching asymptotic values at dGA plating concentrations greater than 400 nM (the underlying correlation coefficients were in the range 0.94–0.99). As the direct binding of dGA alone in the solid phase is saturated at plating concentrations greater than 20 nM (Figure 3a,b), these changes cannot be explained simply by greater concentrations of dGA adsorbed in the solid-phase.

### 3.3. Aqueous-Phase Concentration-Dependent Enhancement of Tau Binding Affinity

Since the binding data exist as a two-dimensional matrix, it is possible to examine binding as a function of the plating concentration of dGA for any given concentration of T40 in the aqueous phase (Figure 2d). The binding data could again be approximated by a similar set of relationships to those shown above. That is, for any fixed concentration of aqueous T40, binding can be expressed as a function of the concentration of solid-phase dGA according to the standard relationship:

*B = B_m2_·[dGA]/(K_d2_ + [dGA])* (4)

Again, the values of the constants *B_m2_* and *K_d2_* vary systematically with the concentration of T40 in the aqueous phase as follows:

*B_m2_ = P_2_·[T40]/(Q_2_ + [T40])* (5)

*K_d2_ = (M_2_/[T40]) + N_2_* (6)

These relationships, depicted in Figure 2e,f, express the asymptotic effects of concentration-dependent changes in aqueous T40 such that the higher the concentration of T40 in the aqueous phase the greater the affinity with which dGA in the solid phase is able to capture T40 from the aqueous phase. The underlying correlation coefficients are in the same range as above. Therefore, the property of concentration-dependent enhancement of binding affinity operates in both solid and aqueous phases.

### 3.4. Comparison of Tau-Tau Binding Binding Affinity Constants in Aqueous and Adsorbed Phases

The formal significance of the empirical parameters *P*, *Q*, *M* and *N*, defined above, can be explained as follows. By substituting the empirical Equations (2) and (3) into Equation (1), the following results were obtained:

[dGA] → ∞,   *B* → *P_1_·[T40]/(N_1_ + [T40])* (7)

[T40] → ∞,   *B* → *P_1_·[dGA]/(Q_1_ + [dGA])* (8)

Likewise, substituting the empirical Equations (5) and (6) into Equation (4) produces the following results:

[T40] → ∞,   *B* → *P_2_·[dGA]/(N_2_ + [dGA])* (9)

[dGA] → ∞,   *B* → *P_2_·[T40]/(Q_2_ + [T40])* (10)

Since Equations (7) and (10) are formally equivalent, N_1_ ≡ Q_2_. These values represent alternative empirical estimates of the common underlying affinity constant for binding of T40 in the aqueous phase to adsorbed dGA in the solid phase when dGA has been plated at a non-limiting concentration. We denote this value as K_d,Aqueous(dGA/T40)_. Dissociation constants are given for the aqueous-phase partner that has been underlined in the pair indicated in parentheses. The similarity of the estimates provided by *N_1_* (22.8 ± 2.8 nM) or *Q_2_* (19.3 ± 0.8 nM) can be seen from Table 2. The mean of these two estimates for K_d,Aqueous(dGA/T40)_ is 21.1 ± 2.9 nM.

Likewise, Equations (8) and (9) are formally equivalent, and N_2_ ≡ Q_1_. These values represent alternative ways of estimating the common underlying affinity constant for the ability of dGA plated in the solid phase to capture T40 from the aqueous phase when T40 has been provided at a non-limiting concentration in the aqueous phase. We denote this value as K_d,Adsorbed(dGA/T40)_. The similarity of the estimates provided by *N_2_* (36.3 ± 21.4 nM) or *Q_1_* (26.6 ± 7.3 nM) can be seen from Table 2. The mean of these two estimates for K_d,Adsorbed(dGA/T40)_ is 31.5 ± 22.6 nM. The difference between the values for K_d,Aqueous(dGA/T40)_ and K_d,Adsorbed(dGA/T40)_ is not statistically significant (*p* = 0.324).

### 3.5. Binding Affinities in the Aqueous Phase Following (Hyper)Phosphorylation of Tau Protein

When recombinant human tau protein was hyperphosphorylated *in vitro*, the K_d,Aqueous(dGA/T40P)_ for T40P binding to adsorbed dGA was 236.5 ± 96.3 nM (Table 2), indicating 11-fold inhibition relative to the non-phosphorylated form, a difference that is significant (*p* = 0.013).

Experiments were also conducted using newborn tau extracted from newborn rat brains. Newborn rat brain tau (“NT”) is an endogenously phosphorylated form of predominantly 3-repeat tau [40,41]. It can be hyperphosphorylated further *in vitro* (“NTP”) by incubation with a crude rat brain extract, as shown for example by the acquisition of immunoreactivity recognized by the phosphorylation-dependent mAb 21/D10 raised against the A68 preparation of sarkosyl-insoluble tau from AD brain [9] (Figure 1c).

**Figure 4:**
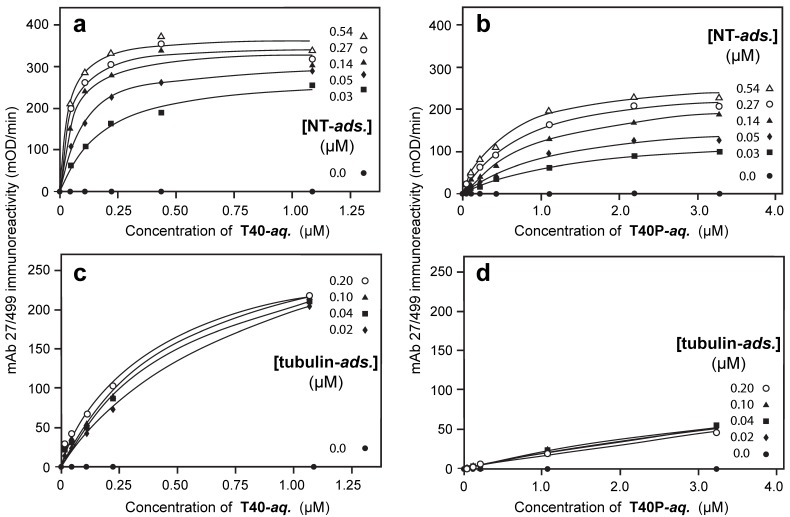
Intrinsic inhibitory effects of phosphorylation. Binding of T40 and T40P in the aqueous-phase to adsorbed neonatal tau (“NT”; (**a**,**b**)) or adsorbed depolymerised bovine tubulin (**c**,**d**) at the plating concentrations indicated. Although NT in the aqueous-phase did not bind to solid-phase dGA (data not shown), binding of T40 (a) to NT was indistinguishable from that seen with dGA in the solid-phase. Binding was inhibited by a factor of 24-fold following *in vitro* hyperphosphorylation of recombinant tau (“T40P”; (b)). Binding of recombinant human tau to depolymerised bovine tubulin (c) was likewise inhibited by a factor of 24-fold following hyperphosphorylation of tau *in vitro* (d). Tau binding was detected using mAb 27/499.

When endogenously phosphorylated NT was incubated in the aqueous-phase with adsorbed dGA, no binding to adsorbed dGA was detected even at concentrations as high as 1 µM (data not shown). However, when NT was adsorbed in the solid-phase of the assay, the binding affinity of T40 was experimentally indistinguishable from its binding to adsorbed dGA (Figure 2a and Figure 4a; Table 2; K_d,Aqueous(NT/T40)_ = 20.8 ± 10.5 nM; *p* = 0.511). Therefore, adsorption of NT to the solid-phase substrate overcomes the inhibitory effect of the endogenous state of phosphorylation in NT hindering its binding to the repeat domain tau fragment in the solid phase and provides the same binding affinity for T40 as the dGA repeat domain fragment.

**Figure 5:**
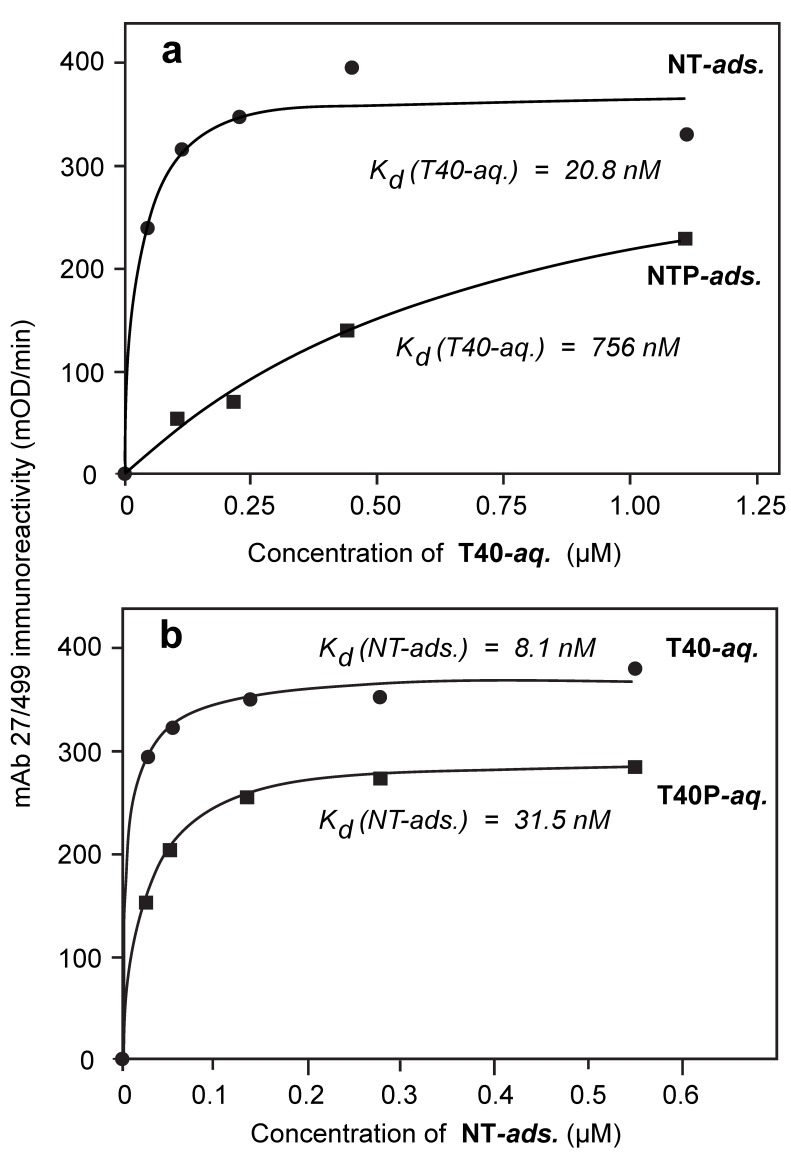
Induced inhibitory effects of tau phosphorylation. (**a**) Representative binding curves for nonphosphorylated T40 to adsorbed neonatal NT or NTP. Binding data is shown only for the case in which adsorbed neonatal tau was present at saturating concentration (>0.54 μM) but, for calculation of the K_d_ values shown here and in Table 2, binding was measured over the full range of NT concentrations shown in Figure 4. Prior hyperphosphorylation of neonatal tau (adsorbed NTP) inhibited binding of T40 in the aqueous-phase by a factor of 36-fold; (**b**) Representative curves for binding of adsorbed NT at saturating concentrations of T40 or T40P (>654 μM and >4362 μM, respectively). The corresponding solid-phase binding constants calculated from data obtained over the full range of aqueous-phase tau concentrations are shown. The effect of hyperphosphorylation of tau in the aqueous-phase is to reduce the binding affinity of the adsorbed species by a factor of 3-fold.

Binding of T40P to adsorbed neonatal tau was inhibited 24-fold relative to binding of T40 to either adsorbed NT (K_d,Aqueous(NT/T40P)_ value 504 ± 146 nM (Table 2); Figure 4b; *p* = 0.00048) or dGA. When newborn rat tau had been hyperphosphorylated prior to plating in the solid phase (NTP), the K_d,Aqueous(NTP/T40)_ value for binding of T40 was 756 ± 228 nM (36-fold inhibition; Table 2; Figure 5a; *p* = 0.00063). The corresponding K_d,Aqueous(NTP/T40P)_ value for binding hyperphosphorylated T40P was 1066 ± 223 nM, indicating 50-fold inhibition (Table 2; *p* < 0.0001).

### 3.6. Binding of Phosphorylated or Non-Phosphorylated Tau in the Aqueous Phase to Depolymerized Tubulin

For comparison, the affinity for binding of tau in the aqueous phase to depolymerized bovine tubulin in the solid phase was K_d,Aqueous(Tub/T40)_ = 403 ± 86 nM. The corresponding K_d,Aqueous(Tub/T40P)_ value for T40P was 9583 ± 3227 nM, indicating a 24-fold inhibition following tau hyperphosphorylation (*p* = 0.0022) (Figure 4).

### 3.7. Solid-Phase Binding Constants in the Presence or Absence of Hyperphosphorylation

As shown above, it is possible to calculate the solid-phase binding constant which describes the affinity with which tau protein in the adsorbed state is able to capture tau protein from the aqueous phase when the latter is available at non-limiting concentration. The affinity of dGA in the solid phase for binding T40P in the aqueous-phase (K_d,Adsorbed(dGA/T40P)_) was 28.7 ± 19.5 nM, despite the 11-fold difference in the corresponding aqueous-phase binding constant seen in the same experiment (Table 2). The similarity in the binding affinity of dGA in the solid-phase for capture of either T40 (K_d,Adsorbed(dGA/T40)_ = 31.5 ± 22.6 nM) or T40P (K_d,Adsorbed(dGA/T40P)_ = 28.7 ± 19.5 nM) in the aqueous phase can be seen in (Table 2; *p* = 0.537).

Similar results were also obtained when full-length newborn rat tau protein was used as the absorbed species, either in the native state of phosphorylation (NT) or following its further hyperphosphorylation *in vitro* (NTP). For NT (K_d,Adsorbed(NT/T40)_ = 8.1 ± 5.2 nM) or NTP (K_d,Adsorbed(NTP/T40)_ = 10.5 ± 3.8 nM) adsorbed in the solid phase, the affinities for capture of T40 in the aqueous-phase at non-limiting concentration (Figure 5b; Table 2) were essentially identical (*p* = 0.356).

The only instances where adsorption in the solid phase did not entirely negate the inhibitory effect of (hyper)phosphorylation in neonatal tau, as seen via the solid-phase binding constant, were those where the aqueous-phase species was itself hyperphosphorylated. Thus, the binding affinity of NT adsorbed in the solid phase for capture of T40P from the aqueous phase was 3-fold less than for T40 in the aqueous phase (Table 2; K_d,Adsorbed(NT/T40P)_ = 31.5 ± 5.1 nM; K_d,Adsorbed(NTP/T40)_ = 10.5 ± 3.8 nM; Figure 5b; *p* = 0.00048). The binding affinity of NTP adsorbed in the solid phase for capture of T40P was 10-fold less than for T40 (Table 2; K_d,Adsorbed(NTP/T40P)_ = 100.4 ± 17.5 nM; K_d,Adsorbed(NTP/T40)_ = 10.5 ± 3.9 nM; *p* < 0.0001).

## 4. Discussion

### 4.1. Features of the Tau-Tau Binding Assays

The present study shows that the tau-tau binding interaction through the repeat domain has several unexpected properties. A particularly useful feature of the assay configuration and analytical methodology we have used in this study is that it provides a means for elucidating binding properties in both solid and aqueous phases. This in turn makes it possible to examine separately the effect on tau-tau binding of prior adsorption to a solid-phase substrate, the effect in both phases of chemical modification of tau by means of phosphorylation, and to compare these properties with corresponding effects on the physiological tau-tubulin binding interaction. This can all be achieved in the same assay configuration and using the same antibody to detect the binding interaction.

All of the binding data can be described in a surprisingly consistent manner by a system of typical Langmuir equations. This in turn implies that the tau-tau binding interaction is competitive, saturable and reversible. For any given set of conditions, binding can be defined quantitatively by a binding constant (K_d_) and a saturation binding value (B_max_). For the tau-tau binding interaction to be described systematically in this way, the following assumptions must be met: (1) for any given set of conditions, individual binding sites operate independently of one another (*i.e.*, there is no positive or negative cooperativity between them); (2) the number of binding sites is finite; and (3) binding is reversible, with on and off rates that are determined entirely by the relative concentrations of the interacting species. The absence of cooperativity is supported by the data fitting well to the Langmuir equation with the Hill coefficient set to one, with correlation coefficients greater than 0.94. The implied reversibility has particular theoretical importance in that it contradicts claims (also based on *in vitro* binding data) that the formation of covalent bonds via cysteine residues is necessary to support tau-tau binding through the repeat domain [42]. This is further supported by a recent report that the formation of fibrils from a Tau208-324 fragment does not depend upon cross-linking between cysteine residues [43].

### 4.2. The Binding Characteristics for Tau in Solid and Aqueous Phases

For any given plating concentration of the repeat-domain dGA fragment in the solid phase, binding of full-length, non-phosphorylated 4-repeat tau (T40) presented in the aqueous phase can be described by characteristic values for K_d1_ and B_m1_. The value for B_m1_, which is the maximum readout of the assay as the concentration of T40 approaches saturation, represents a measure of the maximum number of binding sites provided by dGA in the solid phase at a given plating concentration for capture of T40 from the aqueous phase. The dependency of the K_d1_ of the aqueous species on the plating concentration of dGA in the solid phase indicates that that, at low plating concentrations of solid-phase dGA, T40 binds with low affinity, even when T40 is available at non-limiting concentration in the aqueous phase. As [dGA] → ∞ the K_d1_ value drops to a limiting asymptotic value which we denote N_1_. It can be shown that this asymptotic value is the same as Q_2_, which is the affinity constant calculated from data expressed as dGA capture of T40 when [dGA] → ∞ at varying concentrations of T40 and determined as B_m2_ (*i.e.*, B_max_). N_1_ and Q_2_ therefore represent alternative ways of estimating the same underlying limiting value for K_d,Aqueous (dGA/T40)_ for binding of T40 in the aqueous phase as [dGA] → ∞, for which the mean value is 21.1 ± 2.9 nM.

It is also possible to examine the capture of aqueous phase T40 by solid-phase dGA at a fixed concentration of T40 but at varying concentrations of dGA. For a given concentration of T40 in the aqueous phase, the amount of T40 captured at different concentrations of solid-phase dGA can be described by a similar set of Langmuir equations, each with its apparent K_d2_ and B_m2_ values. The values of K_d2_ decrease with increasing concentrations of T40 to an asymptotic value as [T40] → ∞, which we denote by N_2_. As above, it can be shown that this asymptotic value is formally the same as Q_1_. The empirical parameters N_2_ and Q_1_ therefore represent alternative ways of estimating the same underlying limiting value for K_d,Adsorbed (dGA/T40)_ for capture, by solid phase dGA (underlined), of T40 from the aqueous phase as [T40] → ∞, for which the mean value is 31.5 ± 22.6 nM.

The concentration-dependent enhancement in binding affinity implies that there is an avidity effect, and the phase reciprocity of the phenomenon implies that it operates in both phases. This avidity effect is most likely due to the closer alignment of dGA molecules with increasing concentration, such that each molecule of T40 may interact with more than one molecule of dGA. For this effect to be observed maximally, solid-phase concentrations of dGA must approach 400 nM. This cannot be due to a concentration-dependent increase in the actual amount of dGA on the solid phase, since dGA binding to the plate saturates at concentrations greater than 20 nM. Furthermore, the critical incubation concentration of T40 in the aqueous phase when this effect is observed is only ~20 nM. This disparity in concentrations supports the interpretation that each T40 molecule in the aqueous phase is able to bind to many dGA molecules in the solid phase (*i.e.*, “one-to-many” binding).

It is surprising that the same avidity effect must also operate in the aqueous phase, whereby dGA molecules adsorbed in the solid phase are able to capture T40 from the aqueous phase with higher affinity as the incubation concentration of T40 increases. Again, this is most likely due to a closer alignment or packing with increasing concentration of T40 molecules in the aqueous phase such that each molecule of dGA is able to interact with more than one molecule of T40 in the aqueous phase. This is consistent with the increase in B_m2_ values with increasing concentrations of T40. For this effect to be observed maximally, the incubation concentrations of T40 must approach 250 nM, whereas the critical plating concentration of dGA when this effect is observed is ~30 nM. This again supports the interpretation that avidity is explained by the capacity for one molecule in the solid phase to bind to many in the aqueous phase.

### 4.3. Impact of Phosphorylation on Binding Characteristics

The reciprocity of the aqueous- and solid-phase relationships breaks down when one or both binding partners are hyperphosphorylated. The aqueous-phase binding affinity of T40 to endogenously phosphorylated full-length neonatal tau (NT) in the solid phase is identical to the binding affinity of T40 to dGA in the solid phase. This implies that the dGA fragment encompassing only the phase-shifted version of repeats 2/3/4 contains all that is needed for binding of T40, and that neither provision of a full-length tau molecule in the solid phase not its endogenous state of phosphorylation have any impact on the aqueous-phase binding affinity. However, binding of phosphorylated T40P in the aqueous phase to adsorbed dGA is inhibited 11-fold relative to the binding of T40 to dGA. When NT in the solid phase is hyperphosphorylated to NTP, binding of non-phosphorylated T40 in the aqueous phase was inhibited 36-fold relative to its binding to NT. If, in addition, T40 is phosphorylated to T40P, its binding to NTP in the solid phase is inhibited 50-fold relative to binding of T40 to NT. Thus, whereas provision of NT in its endogenous state of phosphorylation in the solid phase does not enhance capture of T40 from the aqueous phase, any further phosphorylation of either the solid- or aqueous-phase species produces a substantial hindrance to binding.

The inhibitory effect of phosphorylation of the aqueous-phase species on its binding to dGA occurs despite plating of dGA at a concentration which ought otherwise to enhance binding due to the avidity effect described above. T40P molecules in the aqueous phase are inherently less able to bind to dGA in the solid phase. This is most likely due to stabilization of a conformation in the aqueous phase, which impedes access to the critical repeat domain-binding region. The most striking instance of this is the endogenous state of phosphorylation of NT in the aqueous phase, which entirely blocks its ability to bind through the repeat domain even at high concentration (*i.e.*, up to 1 μM). This presumably represents an adaptation preventing initiation of the tau aggregation cascade during a period of intense developmental plasticity. The same phenomenon can be seen to operate for T40 binding to tubulin whereby, in the hyperphosphorylated state, the binding affinity of T40P is reduced by a factor of 24-fold. This is in line with observations previously reported [17,39,44,45].

The inhibitory effect of phosphorylation can be seen even if the species in the aqueous phase is not itself phosphorylated. Thus, as noted above, binding of non-phosphorylated T40 to hyperphosphorylated NTP on the solid phase is inhibited 36-fold relative to its binding to endogenously phosphorylated NT on the solid phase. This suggests that even in the adsorbed state and availability at concentrations where [NTP] → ∞, hyperphosphorylation of neonatal tau prevents it from entering into the alignment/packing interactions in the solid phase required to support the avidity effect discussed above, which is required for high affinity binding of T40.

It is surprising to find that the solid-phase binding constant for dGA is unaffected by the state of phosphorylation of the aqueous-phase species. Thus the values for K_d,Adsorbed (dGA/T40)_ and K_d,Adsorbed (dGA/T40P)_ are indistinguishable, notwithstanding the 11-fold increase in the value of the aqueous-phase binding constant (*i.e.*, K_d,Aqueous (dGA/T40P)_ = 11 × K_d,Aqueous (dGA/T40)_). Since the asymptotic solid-phase binding constant is determined when the concentration of the aqueous-phase species is non-limiting, the implication is that the impediment to alignment/packing caused by phosphorylation has a reversible conformational character that can be overcome at a sufficiently high concentration of the aqueous-phase species. Similarly, the values for K_d,Adsorbed (NT/T40)_ and K_d,Adsorbed (NTP/T40)_ are indistinguishable. That is, provided the species in the aqueous phase (T40) is not phosphorylated, hyperphosphorylation of neonatal tau adsorbed to the solid phase makes no difference to the solid-phase binding constant. Furthermore, the identity of the solid-phase binding constants for binding either T40 or T40P in the aqueous phase implies that phosphorylation does not change the sites in the tau molecule required for high affinity binding. The constancy of the solid-phase binding constants appears at first site to contradict the finding that hyperphosphorylation of neonatal tau in the solid-phase impairs the aqueous-phase binding affinity of T40 by a factor of 36-fold. However, the solid-phase binding constant is determined as [T40] → ∞ in the aqueous phase. In other words, the concentration-dependent alignment or packing of T40 molecules in the aqueous phase required to support the avidity effect is able to occur at a sufficiently high concentration of the aqueous phase species.

### 4.4. Effect of Regions Outside the Repeat Domain on Binding

If the species in the aqueous phase is phosphorylated to T40P, the value for K_d,Adsorbed_ is increased three-fold for NT in the solid phase, and 10-fold for NTP in the solid phase. As discussed above in the case of dGA in the solid phase, phosphorylation of the aqueous-phase species makes no difference to the solid-phase binding constant. A possible explanation is that as either [T40] → ∞ or [T40P] → ∞ in the aqueous phase, the unfavorable conformation produced in the aqueous phase by phosphorylation is overcome, and solid-phase binding at the critical concentration (*i.e.*, ~30 nM) is able to benefit via the one-to-many mechanism underlying avidity. However, when the species in the aqueous phase is phosphorylated (T40P) and the species in the solid phase is either endogenously phosphorylated (NT) or hyperphosphorylated (NTP), some further factor associated with regions outside the repeat domain increases the solid-phase binding constant. This effect must pertain to regions of the molecule outside the repeat domain, since the effect is not seen with dGA in the solid phase. The factor most likely to be responsible is a net charge effect, whereby the presence of phosphate groups in regions outside the repeat domain, in both solid- and aqueous-phase binding partners, simply produce a direct inhibition that is not mediated via conformation.

Regions outside the repeat domain are nevertheless able to contribute strongly to the solid-phase binding affinity. This is based on the observation that, provided the species in the aqueous phase is non-phosphorylated T40, the values of the solid-phase binding constant for NT or NTP are one third that for dGA in the solid phase (*i.e.*, the values for K_d,Adsorbed (NT/T40)_ = 8.1 ± 5.2 nM and K_d,Adsorbed (NTP/T40)_ = 10.5 ± 3.9 nM are one third of K_d,Adsorbed (dGA/T40)_ = 31.5 ± 22.6 nM). Therefore, the additional regions outside the repeat domain appear to provide a further binding advantage in the solid phase. In this configuration, avidity is presumed to be operating in the aqueous phase as [T40] → ∞, permitting one-to-many binding to occur for either NT or NTP at the critical concentration in the solid phase (*i.e.*, ~10 nM). One possible explanation might be considered to relate to the fact that the repeat domain of neonatal tau comprises repeats 1/3/4, whereas the dGA fragment comprises the phase-shifted version of repeat 2/3/4. However, as noted earlier, the aqueous-phase affinity constants for binding of T40 to dGA or NT in the solid phase are identical (*i.e.*, K_d,Aqueous (dGA/T40)_ = 21.1 ± 2.9 nM and K_d,Aqueous (NT/T40)_ = 20.8 ± 10.5 nM). The difference in the repeat domains is therefore unlikely to be the explanation. A more plausible alternative is that binding of regions of the tau molecule outside the repeat domain to the plate further facilitate acquisition of the optimal configuration required for tau-tau binding at the critical solid-phase concentration. For example, in the case of dGA, plate binding and tau-tau binding must occur through the same domain, whereas if NT or NTP is provided, the adsorption interaction may be mediated by domains outside the repeat domain, leaving the repeat domain with greater freedom to associate.

### 4.5. Tau-Tubulin Interactions

In contrast to the complexities of tau-tau binding, the tau-tubulin binding interaction appears to be much simpler. First, no avidity effect was seen. Providing tubulin in the solid phase at higher concentration did not enhance tau-tubulin binding. The value of K_d,Aqueous (Tub/T40)_ remained constant irrespective of the solid-phase concentration of tubulin. Second, as noted already, the effect of phosphorylation of tau was comparable in both tau-tubulin and tau-tau binding interactions. For tau-tubulin binding, phosphorylation produced a 24-fold inhibition following tau phosphorylation. For tau-tau binding, phosphorylation also produced an 11- to 50-fold inhibition of binding, depending on the exact configuration. However, tau-tau binding was favored over tau-tubulin binding by a factor of 19-fold if tau is non-phosphorylated (21.1 ± 2.9 nM vs 403 ± 86 nM, respectively, for T40 binding to dGA or tubulin in the solid phase) and 41-fold if tau is phosphorylated (236.5 ± 96.3 nM *vs.* 9583 ± 3227 nM, respectively, for T40P binding to dGA or tubulin in the solid phase). There is, therefore, no need to invoke phosphorylation as a mechanism to explain detachment of tau from microtubules and the 80-fold redistribution of the tau pool from tubulin-bound to aggregated forms that is seen in AD [1]. Once either full-length tau or the truncated repeat-domain fragment are available bearing conformational changes similar to those produced by adsorption in the solid-phase, the inherent tau-tau binding affinities will drive a progressive redistribution of the tau protein pool away from tubulin-bound towards the aggregated state. We have shown previously, in a biochemical study of progression of tau aggregation in the human brain, that the loss of soluble tau (released from depolymerized microtubules) is not sufficient to explain the accumulation of aggregated tau in the AD brain. Rather, there appears to be increased synthesis of new tau to compensate for the continued loss of soluble tau through aggregation [45]. This continues until a point is reached where the overall rate of aggregation exceeds the ability of the neuron to compensate by synthesis of new tau protein. It is only at this relatively late stage that tau aggregation becomes visible histologically in the form of neurofibrillary tangles, a stage that typically occurs some 20 years after onset of tau aggregation in the neocortex [17,46].

### 4.6. Therapeutic Implications

From this study, it appears that the most important factor required for conversion of tau protein from non-aggregating to aggregating states is to possess a conformational change similar to that which is induced by binding to a solid-phase polyanionic substrate (“PAS”) analogous to the poly(vinyl chloride) plate present in our assay. This process does not depend upon prior post-translational modifications such as hyperphosphorylation (e.g., [12,13]) or proteolytic truncation (e.g., [47,48]) as essential initiating events, nor does the critical pathological interaction require to be stabilized by formation of covalent bonds at cysteine residues as has been suggested [42,49]. Once the pathological solid-phase configuration of the repeat domain has been acquired, further tau aggregation can then be driven by high-affinity binding interactions which are self-replicating and self-amplifying [27]. The resulting aggregate is proteolytically stable and self-propagating, *i.e.*, prion-like [19].

In AD, neurofibrillary lesions first appear in locus coeruleus and entorhinal cortex, followed by hippocampal formation and large parts of the neocortex [50,51]. There is now further evidence from cellular and mouse models that assembled tau can behave like a prion, with the intercellular transfer of tau oligomers that can become self-propagating [52] and are capable of passage of aggregated tau from transgenic mice [53,54,55,56]. Distinct conformers of assembled tau [53,57,58,59] akin to prion strains, may help account for the selective neuronal vulnerability characteristic of specific tauopathies other than AD [60,61]. The active conformations may also offer potential for immunotherapeutic approaches in the treatment of tauopathies [62]. Although there is much yet to be understood about the mechanisms of transmission of tau pathology [60], our findings do not support a direct role for phosphorylation in this process.

From examination of all the binding configurations available for analysis in this study, the lowest value binding constant is K_d,Adsorbed (NT/T40)_ = K_d,Adsorbed (NTP/T40)_ = ~10 nM. This represents the minimum energy configuration required to trigger the tau aggregation cascade, and represents the solid-phase binding constant for full-length neonatal tau (whether or not hyperphosphorylated) bound to a PAS binding to full-length non-phosphorylated adult tau available at non-limiting concentration in the aqueous phase. For reasons of assay consistency required to make comparisons, the only species examined in the solid phase (other than dGA) was neonatal tau, because both lack the human-specific, N-terminal epitope recognized by mAb 27/499 used in the detection of T40 binding. Nevertheless, there is no reason to think that full-length adult tau in the solid phase would behave any differently, given that T40 binding to the truncated dGA fragment from 4-repeat tau and neonatal tau were indistinguishable.

**Figure 6:**
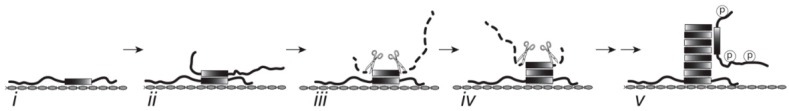
Schematic representation of tau-tau binding. (i) Tau is initially captured by binding to a solid-phase substrate. The identity of the endogenous substrate in the aging brain is unknown, but may be formed from macromolecular complexes which escape normal endosomal/lysosomal processing with advancing age; (ii) The tau bound to a solid-phase substrate is able to capture further full-length tau through a tau-tau binding interaction which has greater affinity (lower energy) than the physiological tau-tubulin binding interaction; (iii) This locks the repeat domain into a proteolytically stable configuration such that proteolytic cleavage of the N- and C-termini leaves behind a characteristic tau fragment restricted to the repeat domain, the core tau unit of the PHF; (iv) The oligomeric form of truncated tau is able to propagate capture and proteolytic processing of further tau, and repeated cycles of binding and proteolysis result in accumulation of PHF-core tau; (v) It is only at a later stage that non-proteolysed, full-length tau molecules are added and these become secondarily phosphorylated. The optimal points of pharmaceutical intervention are therefore blocking the binding of tau to the initiating endogenous substrate or blocking the propagation of tau aggregation cascade with tau aggregation inhibitors.

Based on data from the present study, the “optimal” sequence of events required to initiate pathological aggregation of tau protein is summarized schematically in Figure 6. *In vitro*, tau aggregation can be facilitated by PASs, such as heparin or RNA [30,32,63] or by poly(vinyl chloride), as demonstrated here. The substrate that initiates the capture of tau in AD is not known, but proteins and/or macromolecular complexes that escape normal endosomal/lysosomal processing could provide an endogenous substrate to trigger/nucleate the pathological tau aggregation cascade in the AD brain [19]. In our view, therefore, the search for disease-modifying drugs for AD should be directed not only at inhibitors of tau-tau aggregation, but also on the identification and characterization of critical PASs *in vivo* and the screening for drugs which either block the formation of the substrate or interfere with the interaction between tau and the PAS, which drives downstream tau-tau aggregation.

## 5. Conclusions

We have examined *in vitro* tau-tau binding interactions using a system in which we can observe the impact of tau binding to a solid-phase substrate and determine how phosphorylation modulates this process. Our findings indicate that prior binding of tau to a solid-phase polyanionic substrate is the critical factor in initiating the aggregation of tau protein. This process does not depend upon post-translational modification, such as tau phosphorylation. This has direct implications for targeting pharmaceutical intervention in AD and other tauopathies. Blocking tau aggregation in these disorders requires inhibition of either the capture of tau by, as yet, unidentified protein or macromolecular complexes, or the subsequent tau aggregation cascade.

## Figures and Tables

**Table 1:** Nomenclature for tau proteins.

Tau Protein	Abbreviation	Description
Truncated PHF-core tau	dGA	Bacterial recombinant human tau, residues 297–390 of 2N4R tau isoform [35].
Full-length, non-phosphorylated tau	T40	Bacterial recombinant human tau, 441-amino acid 2N4R tau isoform [33].
Hyperphosphorylated tau	T40P	T40 tau hyperphosphorylated *in vitro* [36].
Neonatal rat tau	NT	Endogenously phosphorylated neonatal rat 3-repeat tau.
Hyperphosphorylated rat tau	NTP	Rat NT hyperphosphorylated *in vitro* [36].

**Table 2:** Saturation aqueous- and solid-phase binding constants (nM) for tau-tau binding.

**Solid-Phase Species**	**Aqueous-Phase Species**	**Solid-Phase Species Non-Limiting**			**Aqueous-Phase Species Non-Limiting**		
		N1	Q2	K_d,Aqeous_	N2	Q1	K_d,Adsorbed_
dGA	T40	22.8 ± 2.8	19.3 ± 0.8	21.1	36.3 ± 21.4	26.6 ± 7.3	31.5
dGA	T40P	252.4 ± 69.8	220.5 ± 66.4	236.5	22.5 ± 17.7	34.9 ± 8.2	28.7
dGA	NT	-*	-	-	-	-	-
NT	T40	18.8 ± 3.4	22.8 ± 9.9	20.8	8.6 ± 5.0	7.6 ± 1.5	8.1
NT	T40P	627 ± 131.0	382.1 ± 64.2	504.5	39.1 ± 4.9	23.8 ± 1.4	31.5
NTP	T40	754 ± 181.0	758.8 ± 138.3	756.4	2.2 ± 1.3	18.8 ± 3.7	10.5
NTP	T40P	969.2 ± 8.8	1163 ± 223	1066.1	86 ± 8.0	114.7 ± 15.6	100.4

N, Q and K_d_ values are defined in the accompanying text. * No binding of NT in aqueous phase to dGA adsorbed in the solid phase.

## References

[1] Harrington C.R., Mukaetova-Ladinska E.B., Hills R., Edwards P.C., Montejo de Garcini E., Novak M., Wischik C.M. (1991). Measurement of distinct immunochemical presentations of tau protein in Alzheimer disease. PNAS.

[2] Drubin D.G., Kirschner M.W. (1986). Tau protein function in living cells. J. Cell Biol..

[3] Alzheimer A. (1907). Über eine eigenartige Erkrankung der Hirnrinde. Allg. Z. Psych. Psych. Gerich. Med..

[4] Nelson P.T., Alafuzoff I., Bigio E.H., Bouras C., Braak H., Cairns N.J., Castellani R.J., Crain B.J., Davies P., Tredici K.D. (2012). Correlation of Alzheimer disease neuropathologic changes with cognitive status: A review of the literature. J. Neuropathol. Exp. Neurol..

[5] Wischik C.M., Harrington C.R., Storey J.M.D. (2014). Tau-aggregation inhibitor therapy for Alzheimer’s disease. Biochem. Pharmacol..

[6] Fodero-Tavoletti M., Furumoto S., Taylor L., McLean C., Mulligan R., Birchall I., Harada R., Masters C., Yanai K., Kudo Y. (2014). Assessing THK523 selectivity for tau deposits in Alzheimer’s disease and non Alzheimer’s disease tauopathies. Alzheimers Res. Ther..

[7] Xia C.F., Arteaga J., Chen G., Gangadharmath U., Gomez L.F., Kasi D., Lam C., Liang Q.W., Liu C.H., Mocharla V.P. (2013). F-18 T807, a novel tau positron emission tomography imaging agent for Alzheimer’s disease. Alzheimer’s Dement..

[8] Cisek K., Cooper G.L., Huseby C.J., Kuret J. (2014). Structure and mechanism of action of tau aggregation inhibitors. Curr. Alzheimer Res..

[9] Lee V.M.-Y., Balin B.J., Otvos L.J., Trojanowski J.Q. (1991). A68: A major subunit of paired helical filaments and derivatized forms of normal tau. Science.

[10] Goedert M., Spillantini M.G., Cairns N.J., Crowther R.A. (1992). Tau proteins of Alzheimer paired helical filaments: Abnormal phosphorylation of all six brain isoforms. Neuron.

[11] Lindwall G., Cole R.D. (1984). Phosphorylation affects the ability of tau protein to promote microtubule assembly. J. Biol. Chem..

[12] Biernat J., Gustke N., Drewes G., Mandelkow E.-M., Mandelkow E. (1993). Phosphorylation of Ser262 strongly reduces binding of tau to microtubules: Distinction between PHF-like immunoreactivity and microtubule binding. Neuron.

[13] Alonso A.D., Zaidi T., Grundke-Iqbal I., Iqbal K. (1994). Role of abnormally phosphorylated tau in the breakdown of microtubules in Alzheimer’s disease. PNAS.

[14] Tseng H.-C., Lu Q., Graves D.J. (1999). Phosphorylated tau can promote tubulin assembly. PNAS.

[15] Buee L., Troquier L., Burnouf S., Belarbi K., van der Jeugd A., Ahmed T., Fernandez-Gomez F., Caillierez R., Grosjean M.E., Begard S. (2010). From tau phosphorylation to tau aggregation: What about neuronal death?. Biochem. Soc. Trans..

[16] Iqbal K., Alonso Adel C., Chen S., Chohan M.O., El-Akkad E., Gong C.X., Khatoon S., Li B., Liu F., Rahman A. (2005). Tau pathology in Alzheimer disease and other tauopathies. Biochim. Biophys. Acta.

[17] Wischik C.M., Edwards P.C., Lai R.Y.K., Gertz H.-J., Xuereb J.H., Paykel E.S., Brayne C., Huppert F.A., Mukaetova-Ladinska E.B., Mena R. (1995). Quantitative analysis of tau protein in paired helical filament preparations: Implications for the role of tau protein phosphorylation in PHF assembly in Alzheimer’s disease. Neurobiol. Aging.

[18] Mukaetova-Ladinska E.B., Garcia-Sierra F., Hurt J., Gertz H.J., Xuereb J.H., Hills R., Brayne C., Huppert F.A., Paykel E.S., McGee M. (2000). Staging of cytoskeletal and b-amyloid changes in human isocortex reveals biphasic synaptic protein response during progression of Alzheimer’s disease. Am. J. Pathol..

[19] Wischik C.M., Lai R.Y.K., Harrington C.R., Avila J., Brandt R., Kosik K.S. (1997). Modelling prion-like processing of tau protein in Alzheimer’s disease for pharmaceutical development. Brain Microtubule Associated Proteins: Modifications in Disease.

[20] Novak M., Jakes R., Edwards P.C., Milstein C., Wischik C.M. (1991). Difference between the tau protein of Alzheimer paired helical filament core and normal tau revealed by epitope analysis of mAbs 423 and 7.51. PNAS.

[21] Bondareff W., Harrington C., Wischik C.M., Hauser D.L., Roth M. (1994). Immunohistochemical staging of neurofibrillary degeneration in Alzheimer’s disease. J. Neuropathol. Exp. Neurol..

[22] Bondareff W., Wischik C.M., Novak M., Roth M. (1991). Sequestration of tau by granulovacuolar degeneration in Alzheimer’s disease. Am. J. Pathol..

[23] Wischik C.M., Novak M., Thøgersen H.C., Edwards P.C., Runswick M.J., Jakes R., Walker J.E., Milstein C., Roth M., Klug A. (1988). Isolation of a fragment of tau derived from the core of the paired helical filament of Alzheimer’s disease. PNAS.

[24] Mena R., Edwards P.C., Harrington C.R., Mukaetova-Ladinska E.B., Wischik C.M. (1996). Staging the pathological assembly of truncated tau protein into paired helical filaments in Alzheimer’s disease. Acta Neuropathol..

[25] Mena R., Edwards P., Pérez-Olvera O., Wischik C.M. (1995). Monitoring pathological assembly of tau and b-amyloid proteins in Alzheimer’s disease. Acta Neuropathol..

[26] Jakes R., Novak M., Davison M., Wischik C.M. (1991). Identification of 3- and 4-repeat tau isoforms within the PHF in Alzheimer’s disease. EMBO J..

[27] Wischik C.M., Edwards P.C., Lai R.Y.K., Roth M., Harrington C.R. (1996). Selective inhibition of Alzheimer disease-like tau aggregation by phenothiazines. PNAS.

[28] Arrasate M., Pérez M., Valpuesta J.M., Avila J. (1997). Role of glycosaminoglycans in determining the helicity of paired helical filaments. Am. J. Pathol..

[29] Friedhoff P., von Bergen M., Mandelkow E.-M., Davies P., Mandelkow E. (1998). A nucleated assembly of Alzheimer paired helical filaments. PNAS.

[30] Goedert M., Jakes R., Spillantini M.G., Hasegawa M., Smith M.J., Crowther R.A. (1996). Assembly of microtubule-associated protein tau into Alzheimer-like filaments induced by sulphated glycosaminoglycans. Nature.

[31] Hasegawa M., Crowther R.A., Jakes R., Goedert M. (1997). Alzheimer-like changes in microtubule-associated protein tau induced by sulfated glycosaminoglycans: inhibition of microtubule binding, stimulation of phosphorylation, and filament assembly depend on the degree of sulfation. J. Biol. Chem..

[32] Pérez M., Valpuesta J.M., Medina M., Montejo de Garcini E., Avila J. (1996). Polymerization of t into filaments in the presence of heparin: the minimal sequence required for t-t interaction. J. Neurochem..

[33] Goedert M., Jakes R. (1990). Expression of separate isoforms of human tau protein: Correlation with the tau pattern in brain and effects on tubulin polymerisation. EMBO J..

[34] Goedert M., Jakes R., Crowther R.A., Six J., Lübke U., Vandermeeren M., Cras P., Trojanowski J.Q., Lee V.M.-J. (1993). The abnormal phosphorylation of tau protein at Ser-202 in Alzheimer’s disease recapitulates phosphorylation during development. PNAS.

[35] Novak M., Kabat J., Wischik C.M. (1993). Molecular characterization of the minimal protease resistant tau unit of the Alzheimer’s disease paired helical filament. EMBO J..

[36] Biernat J., Mandelkow E.-M., Schröter C., Lichtenberg-Kraag B., Steiner B., Berling B., Meyer H., Mercken M., Vandermeeren A., Goedert M. (1992). The switch of tau protein to an Alzheimer-like state includes the phosphorylation of two serine-proline motifs upstream of the microtubule binding region. EMBO J..

[37] Harrington C.R., Edwards P.C., Wischik C.M. (1990). Competitive ELISA for measurement of tau proteins in Alzheimer’s disease. J. Immunol. Meth..

[38] Goedert M., Jakes R., Vanmechelen E. (1995). Monoclonal antibody AT8 recognises tau protein phosphorylated at both serine 202 and threonine 205. Neurosci. Lett..

[39] Schneider A., Biernat J., von Bergen M., Mandelkow E., Mandelkow E.-M. (1999). Phosphorylation that detaches tau protein from microtubules (Ser262, Ser214) also protects it against aggregation into Alzheimer paired helical filaments. Biochemistry.

[40] Kanemaru K., Takio K., Miura R., Titani K., Ihara Y. (1992). Fetal-type phosphorylation of the t in paired helical filaments. J. Neurochem..

[41] Bramblett G.T., Goedert M., Jakes R., Merrick S.E., Trojanowski J.Q., Lee V.M.-Y. (1993). Abnormal tau phosphorylation at Ser396 in Alzheimer’s disease recapitulates development and contributes to reduced microtubule binding. Neuron.

[42] Akoury E., Pickhardt M., Gajda M., Biernat J., Mandelkow E., Zweckstetter M. (2013). Mechanistic basis of phenothiazine-driven inhibition of tau aggregation. Angew. Chem. Int. Ed..

[43] Huvent I., Kamah A., Cantrelle F.-X., Barois N., Slomianny C., Smet-Nocca C., Landrieu I., Lippens G. (2014). A functional fragment of Tau forms fibers without the need for an intermolecular cysteine bridge. Biochem. Biophys. Res. Commun..

[44] Crowther R.A., Olesen O.F., Jakes R., Goedert M. (1992). The microtubule binding repeats of tau protein assemble into filaments like those found in Alzheimer’s disease. FEBS Lett..

[45] Lai R.Y.K., Gertz H.-J., Wischik D.J., Xuereb J.H., Mukaetova-Ladinska E.B., Harrington C.R., Edwards P.C., Mena R., Paykel E.S., Brayne C. (1995). Examination of phosphorylated tau protein as a PHF-precursor at early stage Alzheimer’s disease. Neurobiol. Aging.

[46] Duyckaerts C. (2011). Tau pathology in children and young adults: can you still be unconditionally baptist?. Acta Neuropathol..

[47] Fasulo L., Visintin M., Novak M., Cattaneo A. (1998). Tau truncation in Alzheimer’s disease: Expression of a fragment encompassing PHF core tau induces apoptosis in COS cells. Alzheimer’s Rep..

[48] Fasulo L., Ovecka M., Kabát J., Bradbury A., Novák M., Cattaneo A. (1996). Overexpression of Alzheimer’s PHF core tau fragments: Implications for the tau truncation hypothesis. Alzheimer’s Res..

[49] Schweers O., Mandelkow E.-M., Biernat J., Mandelkow E. (1995). Oxidation of cysteine-322 in the repeat domain of microtubule-associated protein t controls the *in vitro* assembly of paired helical filaments. PNAS.

[50] Braak H., Del Tredici K. (2011). The pathological process underlying Alzheimer’s disease in individuals under thirty. Acta Neuropathol..

[51] Braak H., Braak E. (1991). Neuropathological stageing of Alzheimer-related changes. Acta Neuropathol..

[52] Frost B., Jacks R.L., Diamond M.I. (2009). Propagation of tau misfolding from the outside to the inside of a cell. J. Biol. Chem..

[53] Clavaguera F., Akatsu H., Fraser G., Crowther R.A., Frank S., Hench J., Probst A., Winkler D.T., Reichwald J., Staufenbiel M. (2013). Brain homogenates from human tauopathies induce tau inclusions in mouse brain. PNAS.

[54] De Calignon A., Polydoro M., Suárez-Calvet M., William C., Adamowicz D.H., Kopeikina K.J., Pitstick R., Sahara N., Ashe K.H., Carlson G.A. (2012). Propagation of tau pathology in a model of early Alzheimer’s disease. Neuron.

[55] Liu L., Drouet V., Wu J.W., Witter M.P., Small S.A., Clelland C., Duff K. (2012). Trans-synaptic spread of tau pathology *in vivo*. PLoS ONE.

[56] Clavaguera F., Bolmont T., Crowther R.A., Abramowski D., Frank S., Probst A., Fraser G., Stalder A.K., Beibel M., Staufenbiel M. (2009). Transmission and spreading of tauopathy in transgenic mouse brain. Nat. Cell Biol..

[57] Boluda S., Iba M., Zhang B., Raible K.M., Lee V.M.Y., Trojanowski J.Q. (2015). Differential induction and spread of tau pathology in young PS19 tau transgenic mice following intracerebral injections of pathological tau from Alzheimer’s disease or corticobasal degeneration brains. Acta Neuropathol..

[58] Falcon B., Cavallini A., Angers R., Glover S., Murray T.K., Barnham L., Jackson S., O’Neill M.J., Isaacs A.M., Hutton M.L. (2015). Conformation determines the seeding potencies of native and recombinant tau aggregates. J. Biol. Chem..

[59] Sanders D.W., Kaufman S.K., DeVos S.L., Sharma A.M., Mirbaha H., Li A., Barker S.J., Foley A.C., Thorpe J.R., Serpell L.C. (2014). Distinct tau prion strains propagate in cells and mice and define different tauopathies. Neuron.

[60] Lewis J., Dickson D.W. (2016). Propagation of tau pathology: Hypotheses, discoveries, and yet unresolved questions from experimental and human brain studies. Acta Neuropathol..

[61] Goedert M. (2015). Alzheimer’s and Parkinson’s diseases: The prion concept in relation to assembled Aβ, tau, and α-synuclein. Science.

[62] Pedersen J.T., Sigurdsson E.M. (2015). Tau immunotherapy for Alzheimer’s disease. Trends Mol. Med..

[63] Kampers T., Friedhoff P., Biernat J., Mandelkow E.-M., Mandelkow E. (1996). RNA stimulates aggregation of microtubule-associated protein tau into Alzheimer-like paired helical filaments. FEBS Lett..

