# Erratum: Lai, R.Y.K.; Harrington, C.R.; Wischik, C.M. Absence of a Role for Phosphorylation in the Tau Pathology of Alzheimer’s Disease. *Biomolecules* 2016, *6*, 19

**DOI:** 10.3390/biom6030035

**Published:** 2016-08-12

**Authors:** Robert Y. K. Lai, Charles R. Harrington, Claude M. Wischik

**Affiliations:** 1 Medical Research Council Laboratory of Molecular Biology, Cambridge CB2 0QH, UK; robert.y.lai@btconnect.com; 2 Medical Research Council Cambridge Brain Bank Laboratory, Department of Psychiatry, University of Cambridge, Cambridge CB2 2QH, UK; 3 TauRx Therapeutics Ltd. and School of Medicine, Medical Sciences and Nutrition, University of Aberdeen, Scotland AB25 2ZP, UK; c.harrington@abdn.ac.uk

The authors wish to correct their affiliations in this paper [1] as follows: 

Robert Y. K. Lai ^1,2,†^, Charles R. Harrington ^3^ and Claude M. Wischik ^3,^*

^1^ Medical Research Council Laboratory of Molecular Biology, Cambridge CB2 0QH, UK; robert.y.lai@btconnect.com

^2^ Medical Research Council Cambridge Brain Bank Laboratory, Department of Psychiatry, University of Cambridge, Cambridge CB2 2QH, UK

^3^ TauRx Therapeutics Ltd. and School of Medicine, Medical Sciences and Nutrition, University of Aberdeen, Scotland AB25 2ZP, UK; c.harrington@abdn.ac.uk

* Correspondence: cmw@taurx.com; Tel.: +44-1224-438-550

† Current address: Queen Elizabeth II Hospital, Howlands, Welwyn Garden City, Herts AL7 4HQ, UK.

The authors also wish to acknowledge that the data presented in this study was derived from work undertaken by Robert Y. K. Lai at both institutions of the Medical Research Council towards a PhD thesis submitted to the University of Cambridge in September 1994.
